# What Is Most Suitable for Children With Cystic Fibrosis—The Relationship Between Spirometry, Oscillometry, and Multiple Breath Nitrogen Washout

**DOI:** 10.3389/fped.2021.692949

**Published:** 2022-01-14

**Authors:** Magdalena Postek, Katarzyna Walicka-Serzysko, Justyna Milczewska, Dorota Sands

**Affiliations:** ^1^Cystic Fibrosis Department, Institute of Mother and Child, Warsaw, Poland; ^2^Cystic Fibrosis Centre, Pediatric Hospital, Dziekanów Leśny, Poland

**Keywords:** cystic fibrosis, pulmonary function tests, lung clearance index, impulse oscillometry, spirometry

## Abstract

**Introduction:**

In cystic fibrosis (CF), pathological lung changes begin early in life. The technological progress currently gives many diagnostic possibilities. However, pulmonary function testing in children remains problematic.

**Objectives:**

Our study aimed to correlate the results of impulse oscillometry (IOS) with those of multiple breath nitrogen washout (MBNW) in our pediatric CF population. We also compared those parameters between the groups with and without spirometric features of obturation.

**Methods:**

We collected 150 pulmonary function test sets, including spirometry, IOS, and MBNW in patients with CF aged 12.08 ± 3.85 years [6–18]. The study group was divided into two subgroups: IA (without obturation) and IB (with obturation). We also compared Sacin, Scond, and oscillometry parameters of 20 patients aged 14–18 years who reached the appropriate tidal volume (VT) during MBNW.

**Results:**

Statistical analysis showed a negative correlation between lung clearance index (LCI) and spimoetric parameters. Comparison of subgroups IA (*n* = 102) and IB (*n* = 48) indicated a statistically significant difference in LCI (*p* < 0.001) and FEV1z-score (*p* < 0.001), FEV1% pred (*p* < 0.001), MEF25z-score (*p* < 0.001), MEF50 z-score (*p* < 0.001), MEF75 z-score (*p* < 0.001), R5% pred (*p* < 0.05), and R20% pred (*p* < 0.01). LCI higher than 7.91 was found in 75.33% of the study group, in subgroup IB—91.67%, and IA−67.6%.

**Conclusions:**

LCI derived from MBNW may be a better tool than IOS for assessing pulmonary function in patients with CF, particularly those who cannot perform spirometry.

## Introduction

Pathological changes in the lungs that contribute to a reduction in the quality of life and an increase in mortality appear reasonably early in patients with cystic fibrosis (CF). Despite technological advances and available devices, pulmonary function testing in children remains problematic. The challenges are proper assessment and timely intervention, which are essential to delaying and minimizing disease progression. A gradual decrease in lung function is associated with some physiological factors such as airway obstruction and ventilation heterogeneity (1, 2). These changes should be able to be detected in lung function tests. Not all available techniques are useful due to the difficulties associated with the lack of cooperation in children. Spirometry is considered to be the gold standard of lung function measurements in children older than 6 years and adults (3). This test is routinely used to assess lung function in children with CF. However, spirometry may not be feasible during acute exacerbations and in children with deteriorating lung function. They may not be able to perform spirometry as per standard guidelines; it requires forced expiratory maneuvers (4). It has also been shown that spirometry may not be very sensitive in detecting mild to moderate lung damage in children with CF (5). What is more, during spirometry, a quite large amount of aerosol is generated, which is essential in the coronavirus disease 2019 era (6). Hence, there is a need for a lung function test that is sensitive enough to pick up early abnormalities and that can also be performed easily in preschool children and children with advanced bronchopulmonary disease, who are unable to perform spirometry (4). In our article, we tried to correlate the parameters obtained during the performance of pulmonary function tests using various techniques. We also tried to find a more suitable technique that would better complement the spirometry test for patients who are unable to perform it properly. This is a very important issue in routine clinical practice in CF centers.

The impulse oscillometry (IOS), which is a variant of forced oscillation technique, described by Dubois over 50 years ago, allows evaluation of the mechanical properties of the respiratory system with a minimal need for patient cooperation (7, 8). The IOS technique uses pressure oscillations at a standard square pressure wave, at a frequency of 5 Hz from which all other applied frequencies are derived using spectral analysis. IOS measures the airway impedance (Zrs), which is comprised of two components: resistance (Rrs), which is the real part, and the imaginary part, which is the reactance (Xrs). The resistance reflects the relationship between the applied pressure and the resultant flow. It represents the total respiratory system resistive properties (extrathoracic and intrathoracic airways, lung parenchyma, and chest wall). The resistance at low frequencies (i.e., 5 Hz) reflects the total airway resistance, whereas the resistance at high frequencies (i.e., 20 Hz) reflects large airway resistance. The difference between resistance at 5 Hz and resistance at 20 Hz reflects the small airway resistance. The reactance is a component of impedance that encompasses the capacitive and inertive properties of the lung. It reflects the elasticity of the lung and is negative in sign. Other important parameters in IOS are frequency at which the reactance crosses zero is called resonant frequency (Fres) and AX, which represents the sum of all reactance components at all frequencies before the resonant frequency (6, 9). IOS can differentiate between small and large airway obstruction and is more sensitive than spirometry for peripheral airway disease. It has been used to study various respiratory disorders, especially asthma, and is suitable for measuring bronchodilatory response and bronchoprovocation testing (8). IOS has also been studied in other respiratory diseases such as chronic obstructive pulmonary disease, interstitial lung disease, and supraglottic stenosis (10).

The second technique, which could be helpful, is multiple breath nitrogen washout (MBNW). The lung clearance index (LCI), which is derived from MBNW, is another evolving sensitive tool to assess lung function in children (4). MBNW was introduced in the mid-1950s, but years later, it was put into clinical use. Unfortunately, the performance of MBNW is also much harder for people who cough a lot (pulmonary exacerbations, worse lung disease) and have inflamed airways, which is due to the dry air that they have to inhale during the measurement. Recent years have brought several studies on the IOS technique. In this method, sound waves are superimposed on normal tidal breathing, and the disturbances in flow and pressure caused by the external waves are used to calculate parameters describing the resistance to airflow and reactive parameters that mostly relate to efficient storage and return of energy by the lung (8).

Spirometry, in contrast to IOS or MBNW, is associated with a forced expiration, during which flow restrictions are measured. In IOS, resistance (Rrs) and airway reactance (Xrs) can be measured during tidal breathing. It seems that MBNW and probably IOS are more interesting in those patients with normal spirometry. Therefore, it is worth considering how these techniques are correlated with each other. Data on the comparison of IOS and MBNW parameters with spirometry results in children with CF are limited (4, 10–16).

In our study, we tried to correlate the parameters detected during spirometry, IOS, and MBNW to assess their usefulness in evaluating lung function in children with CF. We also compared those parameters between the groups with and without spirometric features of obturation.

This study was presented in poster form at the European Cystic Fibrosis Society Conference in Liverpool on June 6, 2019.

## Materials and Methods

We conducted a retrospective observational study in Cystic Fibrosis Center in Dziekanow Lesny from March 2017 to June 2019. CF was diagnosed in patients based on clinical features, sweat chloride measurements, and genetic tests according to the current diagnostic criteria (17, 18). Data regarding genotype and medical history were obtained from the clinical records. Patients who were able to perform spirometry, IOS, and MBNW with single-use mouthpieces met the eligibility criteria. The exclusion criteria were lack of cooperation, pulmonary exacerbation, severe clinical condition precluding a patient from performing MBNW, e.g., dyspnea, hemoptysis, and other severe complications of CF.

The protocol was approved on January 10, 2019, by the local ethics committee at the Institute of Mother and Child in Warsaw (opinion number 4/2019). After obtaining approval from the local ethics committee, the participants, and caregivers gave their informed consent for the use of their test results in the study.

All patients were divided into two subgroups depending on the results of the index FEV1/FVC z-score (ratio of forced expiratory volume in 1 s and vital capacity). Group IA (*n* = 102) did not present any evidence of obstruction, and group IB (*n* = 48) showed the features of obstruction in spirometry (FEV1/FVC z-score < −1.65). Criteria for obstruction were established in accordance with the recommendations of the Polish Pulmonary Society in 2005 (19) and based on the guidelines of ERS 1993 and Quarnjer 1989 (20).

To assess inhomogeneity in the acinar (Sacin) and conducting (Scond) airway regions, in the second part of the research, 20 patients with CF aged 15–18 years were extracted from the main group. Those patients were chosen due to their ability to perform MBNW and other tests correctly and to reach the appropriate tidal volume (VT) values required in the standardization documents. In this group, patients performed at least three acceptable and repeatable tests in MBNW, oscillometry, and spirometry. All tests were performed in the morning, starting from IOS, next MBNW, and spirometry. Before the tests, each patient performed the drainage.

We also created two subgroups based on the normal LCI values for healthy children: equal or below (group IC) and above (group ID), a cutoff point of 7.91 (21). This part of the study aimed to analyze the correlation between LCI and parameters such as MEF25, MEF50, MEF75 (maximal expiratory flow at 25, 50, and 75% of FVC), R20, R5 (resistance at 20 and 5 Hz), X5 (reactance at 5 Hz), Ax (area of reactance), and Fres (resonat frequency) in these subgroups.

### Pulmonary Function Measurements

All tests were performed according to the American Thoracic Society and European Respiratory Society guidelines (22–24). All the tests were performed on the same day. Measurements of spirometry and IOS were conducted using Vyntus IOS, Jaeger system (CareFusion, Hochberg, Germany). MBNW tests were performed using Exhalyzer D (EcoMedics AG, Duernten, Switzerland, software version 3.2.0).

Spirometry maneuvers were performed according to the American Thoracic Society and European Respiratory Society guidelines until three repeatable and acceptable measurements were achieved. The spirometer was calibrated every day before measurements with a 3-L syringe. Patients performed spirometry in a sitting position. They would have to achieve at least three reproducible attempts. Test results were considered reproducible if the difference between the two largest values of forced vital capacity FVC was <0.150 L, and the difference between the two largest forced expiratory volume in 1 s (FEV1) values was <0.150 L. The test was considered to be carried out correctly if it met other criteria such as back extrapolated volume (BEV) <5% of FVC or 0.100 L, no cough in the first second of expiration, no glottic closure after 1 s of expiration, no evidence of obstructed mouthpiece and/or spirometer, no evidence of a leak, reaching expiratory plateau <0.025 L in the last 1 s of expiration, or expiratory time > 15 s. We use reference values from Quanjer et al. (25). Reference values from Zapletal et al. (26) were used for MEFs.

The environmental conditions in Exhalyzer D were updated daily. Flow and gas calibrations were performed before measurements. MBNW tests were carried out using single-use mouthpieces and nose clips. Patients performed at least three attempts. The calculations included results from a minimum of two correctly performed and repeatable trials (24). Acceptable maneuvers were defined by the software and also based on the operator's observation of the subject's behavior during testing. Quality control of the study concerned an adequate starting and end-tidal inert gas concentration, stability over 30 s, and regular breathing. We also made sure that there was no evidence of significant trapped gas release with larger breaths, apneas (may significantly decrease functional residual capacity), or sighs (may significantly elevate functional residual capacity) (24).

Oscillometry is potentially affected by upper airway artifacts in the form of swallows, vocal cord closures, coughs, incorrect positioning of the tongue, or mouth leaks; therefore, each test was couched by a technician. According to recommendations, the three replicates used to derive indices had a coefficient of variability of Rrs of ≤15% in children, at the lowest oscillation frequency (27, 28).

### Statistical Analysis

Statistical analysis consisted of calculating means and standard deviations. We used the Spearman test to find a correlation between parameters from spirometry, oscillometry, and MBNW. Analyses were performed for the whole group and for subgroups divided according to spirometric criteria of obstruction, i.e., FEV1/FVC z-score < −1.65 and also for subgroups depending on the LCI value (IC where LCI ≤ 7.91 and ID > 7.91). All values expressed as z-scores were based on sex- and age-specific regression equations. The normality of the data distributions was determined by the Shapiro–Wilk test and graphical analysis. The homogeneity of variance was examined using the Brown–Forsythe test. In the analysis of relationships between unpaired quantitative variables for which no normal distribution was obtained, the non-parametric Mann–Whitney was used. In the analysis of relationships between unpaired quantitative variables, for which a normal distribution and homogeneity of variance were obtained, the Student's *T*-test was used for unpaired samples. The level of statistical significance was set at *p* < 0.05. Data were analyzed with STATISTICA version 13.3.

## Results

### Characteristic of the Study Population

We examined 150 children with CF and adolescents aged 6–18 years who were enrolled (12.08 ± 3.85; 69 males; 81 females). The study group included 61 people with the “*F508del /F508del”* genotype, 72 people with the “*F508del*/other” genotype, and 17 people with mutations specified in the CFTR2 database as pathogenic other than *F508del*. Table 1 shows the biometric characteristics of the whole study group and the main spirometric, MBNW, and IOS parameters. There is also a description of the subgroups.

**Table 1:** CF patients study group and subgroups characteristics: subgroup IA without spirometric features of obturation, IB with obturation features, IC [LCI (≤7.91)] and ID (LCI (>7.91)].

**Parameter**	**Group**	**Subgroup IA**	**Subgroup IB**	**Subgroup IC**	**Subgroup ID**
*N*	150	102	48	36	114
AGE	12.08 ± 3.85	11.74 ± 3.94	12.80 ± 3.57	10.2 ± 3.86	12.47 ± 3.77
FEV_1_ %pred	89.51 ± 17.29	96.26 ± 14.26	75.14 ± 14.17	99.89 ± 14.88	86.23 ± 16.75
FEV_1_ z-score	−0.90 ± 1.49	−0.31 ± 1.20	−2.14 ± 1.26	0.01 ± 1.26	−1.18 ± 1.45
FVC % pred	98.47 ± 14.47	99.32 ± 14.10	96.67 ± 15.22	103.31 ± 15.18	96.95 ± 13, 96
FVC z-score	−0.16 ± 1.25	−0.08 ± 1.19	−0.33 ± 1.35	0.25 ± 1.26	−0.29 ± 1.22
FEV_1_%FVC %	90.38 ± 11.27	96.49 ± 6.06	77.42 ± 8.45	96.67 ± 9.42	88.40 ± 11.12
FEV_1_%FVC z-score	−1.18 ± 1.47	−0.43 ± 0.92	−2.75 ± 1.15	−0.34 ± 1.15	−1.44 ± 1.47
MEF_25_ %pred	−1.39 ± 1.69	88.14 ± 36.70	32.96 ± 16.12	99.81 ± 46.12	61.58 ± 34.18
MEF_25_%z-score	70.82 ± 40.70	−0.59 ± 1.24	−3.13 ± 1.12	−0.32 ± 1.60	−1.73 ± 1.57
MEF_50_ z–score	−0.77 ± 1.75	0.11 ± 1.15	−2.63 ± 1.31	0.28 ± 1.40	−1.10 ± 1.73
MEF_50_ % pred	87.44 ± 33.40	103.99 ± 24.82	52.27 ± 18.87	108.31 ± 30.30	80.85 ± 32.69
LCI	10.16 ± 3.22	9.04 ± 2.19	12.52 ± 3.75	7.03 ± 0.60	11.15 ± 3.07
${\text{S}}_{\text{acin}}^{*}$VT	0.12 ± 0.09	0.10 ± 0.07	0.167 ± 0.11	0.03 ± 0.02	0.14 ± 0.09
${\text{S}}_{\text{cond}}^{*}$VT	0.06 ± 0.03	0.05 ± 0.03	0.08 ± 0.03	0.07 ± 0.07	0.07 ± 0.03
R5%pred	114.89 ± 41.83	110.95 ± 43.14	123.25 ± 37.96	107.61 ± 50.64	117.18 ± 38.60
R20% pred	110.55 ± 28.80	107.10 ± 30.7	117.87 ± 22.90	*108, 67* ± *31, 19*	111.14 ± 28, 12
R5–R20 [kPa/(l/s)]	0.12 ± 0.11	0.12 ± 0.12	0.11 ± 0.07	0.12 ± 0.12	0.12 ± 0.10
X5 %pred	123.88 ± 85.02	114.65 ± 72.54	143.48 ± 105.03	107.11 ± 72.92	129.17 ± 88.13
X20%pred	27.51 ± 125.40	34.26 ± 133.41	13.17 ± 106.28	36.42 ± 133.54	24.70 ± 123.20
F_res_%pred	122.34 ± 42.60	117.89 ± 40.13	131.79 ± 46.46	113.56 ± 40.89	125.11 ± 42.93
AX	1.18 ± 1.05	1.18 ± 1.10	1.18 ± 0.95	1.18 ± 1.11	1.18 ± 1.03
Hight [m]	1.49 ± 0.19	1.48 ± 0.20	1.52 ± 0.18	1.45 ± 0.21	1.51 ± 0.18
HIGHT z–score	0.23 ± 0.99	0.31 ± 1.02	0.05 ± 0.93	0.39 ± 1.28	0.17 ± 0.88
WEIGHT [kg]	42.30 ± 15.49	42.05 ± 16.35	42.82 ± 13.64	36.12 ± 15.38	43.30 ± 15.46
WEIGHT z–score	0.00 ± 1.03	0.12 ± 1.03	−0.28 ± 0.99	0.005 ± 1.13	−0.006 ± 1.00
BMI	18.29 ± 3.16	18.42 ± 3.37	18.02 ± 2.65	17.82 ± 2.91	18.44 ± 3.23
BMI z–score	−0.14 ± 1.04	−0.02 ± 1.03	−0.4 ± 1.03	−0.26 ± 1.16	−0.10 ± 1.00

### Pulmonary Function Measurements

#### Correlation Between Lung Function Parameters of Subgroups: With and Without Spirometric Features of Obstruction

In our study, we compared subgroups of patients with and without spirometric features of obstruction. We found a statistically significant difference between the values of LCI (*p* < 0.001), FEV1z-score (*p* < 0.001), FEV1% pred (*p* < 0.001), MEF25z-score (*p* < 0.001), MEF50 z-score (*p* < 0.001), MEF75 z-score (*p* < 0.001), R5% pred (*p* < 0.05), and R20% pred (*p* < 0.01). There was no statistically significant difference in AX, Fres, Fres% pred, R5, and X5 and X20 in both subgroups.

What is more, a correlation between LCI and FEV1/FVC %pred and FEV1/FVC z-score was found only in the group without obturation (rSpearman = −0.34 and rSpearman = −0.34). In the case of the study of the correlation of parameters from IOS measurements and spirometry, correlations were observed in both groups. We compared parameters such a R5 z-score, R5 % pred, R20 %pred, R20 z-score, X5%pred, X5 z-score, R5-R20, Ax and Fres, Fres %pred with FEV1 %pred, FEV1 z-score, FEV1/FVC %pred, FEV1/FVC z-score, MEF25 %pred, MEF25 z-score, MEF50 %pred, MEF50 z-score, MEF75%pred, and MEF75 z-score. We found some correlations in both groups but not as much strength as in the case of LCI and IOS. We presented the results in Table 2. In addition, we observed a significant negative correlation between LCI and MEF25 %pred, MEF25 z-score, MEF50 %pred, MEF50 z-score, R20Hz, and R5 %pred. Studies on the correlation between LCI and reactance at 5 and 20 Hz showed mostly no relationship between these parameters in patients with CF. Only the X5% pred parameter, in this case, shows a weak correlation with LCI (rSpearman = 0.32). There were no significant correlations between LCI, X20Hz, and R5-R20 Hz parameters in both groups. All significant correlations between lung function test parameters are shown in Table 3.

**Table 2:** Correlations between IOS and spirometry parameters in subgroups: subgroup IA without spirometric features of obstruction and IB with obstruction features.

**Parameter**	**Group I A** r Spearman^*^	**Group I B** r Spearman^*^	**Parameter**	**Group I A** r Spearman^*^	**Group I B** r Spearman^*^
R20 %pred vs. FEV1 %pred	−0.26	−0.35	R20 %pred vs. FEV1 z–score	−0.26	−0.34
R5 %pred vs. FEV1 %pred	−0.33	−0.36	R5 %pred vs. FEV1 z–score	−0.34	−0.33
X5 %pred vs. FEV1 %pred	−0.36	−0.29	X5 %pred vs. FEV1 z–score	−0.37	–
AX vs. FEV1 %pred	–	−0.33	AX vs. FEV1 z–score	–	−0.33
Fres vs. FEV1 %pred	–	−0.33	Fres vs. FEV1 z–score	–	−0.33
Fres %pred vs. FEV1 %pred	–	−0.40	Fres %pred vs. FEV1 z–score	–	−0.38
R5%pred vs. FVC% pred	−0.25	–	R5%pred vs. FVC% z–score	−0.25	–
X5 % pred vs. FVC %pred	−0.35	–	X5 % pred vs. FVC z-score	−0.35	–
R5 %pred vs. FEV1/FVC %pred	−0.22	−0.33	R5 %pred vs. FEV1/FVC z–score	−0.22	–
R5–R20 vs. FEV1/FVC %pred	–	−0.39	R5–R20 vs. FEV1/FVC z-score	–	−0.34
X20 %pred vs. FEV1/FVC %pred	–	0.31	X20 %pred vs. FEV1/FVC z-score	–	–
AX vs. FEV1/FVC %pred	–	−0.39	AX vs. FEV1/FVC z–score	–	−0.37
Fres vs. FEV1/FVC %pred	–	−0.41	Fres vs. FEV1 /FVC z–score	–	−0.39
Fres %pred vs. FEV1/FVC %pred	–	−0.37	Fres %pred vs. FEV1 /FVC z–score	–	−0.29
R5-R20 vs. MEF25 %pred	–	−0.36	R5–R20 vs. MEF25 z–score	–	–
AX vs. MEF25 %pred	–	−0.35	AX vs. MEF25 z–score	–	−0.29
R5%pred vs MEF25 % pred	−0.30	–	R5%pred vs. MEF25 z–score	−0.32	–
R20% pred vs. MEF25 %pred	−0.23	–	R20% pred vs. MEF25 z-score	−0.24	–
X5 %pred vs. MEF25 %pred	−0.38	–	X5 %pred vs. MEF25 %pred	−0.38	–
Fres vs. MEF25 %pred	–	−0.34	Fres vs. MEF25 z–score	–	–
Fres % pred vs. MEF25 %pred	−0.23	−0.36	Fres % pred vs. MEF25 z-score	−0.24	–
R20 %pred vs. MEF50 % pred	−0.21	−0.36	R20 %pred vs. MEF50 z-score	–	−0.35
R5 %pred vs. MEF50 % pred	−0.25	−0.31	R5 %pred vs. MEF50 z-score	−0.23	−0.29
R5-R20 vs. MEF50 % pred	–	−0.34	R5-R20 vs. MEF50 z–score	–	−0.29
X5%pred vs. MEF50 %pred	−0.33	–	X5%pred vs. MEF50 z-score	−0.33	–
X20 vs. MEF50 % pred	–	0.30	X20 vs. MEF50 % z-score	–	–
AX vs. MEF50 % pred	–	−0.40	AX vs. MEF50 z–score	–	−0.36
Fres vs. MEF50 % pred	–	−0.40	Fres vs. MEF50 z–score	–	−0.36
Fres %pred vs. MEF50 % pred	–	−0.34	Fres %pred vs. MEF50 z-score	–	−0.30
R5-R20 vs. MEF 75 %pred	−0.34	−0.44	R5-R20 vs. MEF75 z-score	−0.33	−0.45
X20 % pred vs. MEF 75 %pred	0.34	0.39	X20 % pred vs. MEF z-score	0.34	0.39
AX vs. MEF 75 %pred	−0.41	−0.52	AX vs. MEF 75 z–score	−0.41	−0.53
Fres vs. MEF 75 %pred	−0.42	−0.55	Fres vs. MEF z–score	−0.42	−0.55
Fres % pred vs. MEF 75 %pred	−0.29	−0.34	Fres % pred vs. MEF 75 z-score	−0.29	−0.34

* *p < 0.05*.

*“–”, no statistically important correlation was found*.

**Table 3:** Correlation between lung function parameters of CF patient subgroups: subgroup IA without spirometric features of obstruction and IB with obstruction features.

	**r Spearman**	**p Spearman**
**Subgroup IA**		
LCI vs. FEV1/FVC %pred	−0.34	<0.05
LCI vs. FEV1/FVC z-score	−0.34	<0.05
LCI vs. MEF_25_ z-score	−0.46	<0.05
LCI vs. MEF_25_ %pred	−0.45	<0.05
LCI vs. MEF_50_%pred	−0.32	<0.05
LCI vs. MEF_50_ z-score	−0.32	<0.05
LCI vs. R5 %pred	0.21	<0.05
LCI vs. R20	−0.24	<0.05
LCI vs. X5%pred	0.32	<0.05
**Subgroup IB**		
LCI vs. MEF_25_ z-score	−0.37	<0.05
LCI vs. MEF_50_%	−0.35	<0.05
LCI vs. MEF_50_ z-score	−0.37	<0.05
LCI vs. R5	−0.29	<0.05
LCI vs. R5 %pred	0.34	<0.05
LCI vs. R20	−0.35	<0.05
LCI vs. X5%pred	0.44	<0.05

#### Correlation Between LCI, Spirometry, and Impulse Oscillometry Parameters in Subgroups: With LCI ≤ 7.91 and LCI > 7.91

Based on normal values for healthy children (21), we distinguished that 75.33% of all enrolled patients had LCI above the upper reference limit for healthy children of 7.91 accordingly, 37 patients with LCI ≤ 7.91 (group IC), and 113 patients with LCI > 7.91 (group ID). Moreover, in the group with confirmed obstruction (subgroup IB), LCI values were above 7.91 in a greater percentage of children (91.67%) than in the group without obturation (subgroup IA; 67.6%). The analysis of LCI values showed that in the group of children with normal LCI (≤7.91), there was no significant correlation with the main spirometry and oscillometry parameters. When LCI is above 7.91, this correlation is statistically significant.

We observed statistically significant differences in parameters such as FEV1%pred (*p* < 0.001), FEV1 z-score (*p* < 0.001), FVC%pred (*p* = 0.020), FVC z-score (*p* = 0.030), FEV1/FVC%pred (*p* < 0.001), FEV1/FVC z-score (*p* < 0.001), MEF25%pred (*p* < 0.001), MEF25z-score (*p* < 0.001), MEF50%pred (*p* < 0.001), MEF50z-score (*p* < 0.001), MEF75%pred (*p* = 0.041), MEF75z-score (*p* = 0.042), and R5%pred (*p* = 0.030) in groups IC (LCI ≤ 7.91) and ID (LCI > 7.91), which we presented in Table 4. Correlation between LCI, spirometry, and IOS parameters are presented in Table 5. These correlations were observed only in group ID (LCI > 7.91).

**Table 4:** Statistically significant diferences in spirometry and IOS parameters in subgroups: subgroup IC (LCI ≤ 7.91) and subgroup ID (>7.91).

	**Subgroup IC (LCI ≤7.91)**	**Subgroup ID (>7.91)**	***P*** **-value**
FEV1%pred	99.89 ± 14.88	86.23 ± 16.75	<0.001
FEV1 z-score	0.01 ± 1.26	−1.18 ± 1.45	<0.001
FVC %pred	103.31 ± 15.18	96.95 ± 13.96	0.020
FVC z-score	0.25 ± 1.26	−0.29 ± 1.22	0.030
FEV1/FVC%pred	96.67 ± 9.42	88.40 ± 11.12	<0.001
FEV1/FVC z-score	−0.34 ± 1.15	−1.44 ± 1.47	<0.001
MEF25%pred	99.81 ± 46.12	61.58 ± 34.18	<0.001
MEF25 z-score	−0.32 ± 1.60	−1.73 ± 1.57	<0.001
MEF50%pred	0.28 ± 1.40	−1.10 ± 1.73	<0.001
MEF50 z-score	108.31 ± 30.30	80.85 ± 32.69	<0.001
MEF75%pred	92.65 ± 21.50	83.78 ± 21.65	0.041
MEF75 z-score	−0.64 ± 1.38	−1.22 ± 1.47	0.042
R5%pred	107.61 ± 50.64	117.18 ± 38.60	0.030

**Table 5:** Correlation between LCI, spirometry, and IOS parameters in subgroup ID (LCI > 7.91).

**Parameter**	***r*** **Spearman**	***p*** **Spearman**
LCI vs. FEV_1_%pred	−0.62	<0.05
LCI vs. FEV_1_z-score	−0.61	<0.05
LCI vs. FVC%pred	−0.41	<0.05
LCI vs. FEV_1_/FVC%pred	−0.52	<0.05
LCI vs. FEV_1_/FVC% z-score	−0.51	<0.05
LCI vs. MEF_25_%pred	−0.62	<0.05
LCI vs. MEF_25_%z-score	−0.64	<0.05
LCI vs. MEF_50_%pred	−0.55	<0.05
LCI vs. MEF_50_%z-score	−0.55	<0.05
LCI vs. MEF_75_%pred	−0.33	<0.05
LCI vs. MEF_75_%z-score	−0.33	<0.05
LCI vs. R20	−0.26	<0.05
LCI vs. R20%pred	−0.26	<0.05
LCI vs. R5	−0.25	<0.05
LCI vs. R5%pred	0.36	<0.05
LCI vs. X5%pred	0.36	<0.05

#### Correlation Between Sacin, Scond, and IOS and Spirometry Parameters

Based on the analysis performed on 20 persons, no significant correlation was found between the Sacin and IOS parameters. We found correlation between Scond and R5Hz, R20Hz (rSpearman = 0.56 and rSpearman = 0.55), and spirometric parameters such as FEV1/FVCz-score (rSpearman = −0.62), MEF25 (rSpearman MEF25%pred = −0.69; rSpearman MEF25z-score = −0.75), MEF50 (rSpearman MEF50%pred = −0.60, rSpearman MEF50z-score = −0.58), and MEF75 (rSpearman MEF75%pred = −0.46, rSpearman MEF75z-score = −0.45).

## Discussion

During the last few decades, conventional spirometry was the method of choice for assessing respiratory status in patients with CF. However, this test is not applicable in small children, as adequate coordination and cooperation are required. Additionally, spirometry is not sensitive enough for evaluating early CF lung disease (29, 30). For these reasons, scientific interest in other non-invasive methods such as IOS and MBNW has been reintroduced over the last 15 years (31). These techniques seem to be alternative diagnostic tools to monitor lung function.

The main parameter of MBNW-LCI was presented to be more sensitive than spirometry or plethysmography parameters in monitoring early lung disease in children (31, 32). LCI values increase with lung disease severity (33). On the other hand, it is a quite difficult test for patients with exacerbations because dry air may increase coughing. For this reason, oscillometry seems to be the simplest to be performed by all patients, but it is worth considering whether its results are sufficiently related and correspond to other techniques. In this paper, we tried to investigate the relationship between oscillometry, spirometry, and MBNW results in 150 children with CF aged 6–18 years. Based on our analysis of the observed results, we showed that LCI tends to increase in patients with lower values of spirometry parameters such as FEV1 z-score and FVC z-score. We also received statistically significant moderate negative correlations between LCI and MEF25z-score, MEF50z-score. However, we did not document a significant correlation between LCI and reactance at 5 and 20 Hz or R5-R20. Presumably, the presence of homogenic central obturation does not influence reactance significantly.

There is a limited number of studies with patients with CF of which the aim was to evaluate and monitor pulmonary functions with IOS (4, 10–16). The relationship between spirometry and IOS results in children with CF is contradictory. Toprak et al. (10), in the study comparing clinical severity scores and classic spirometry with IOS results and thoracic high-resolution computed tomography scores in children with CF, observed that patients with FEV1 below 80% exhibited significantly higher (resistance) R5 and R10 values and significantly lower (reactance) X5 values on IOS. Moreau et al. (11) evaluated the relationship between IOS parameters and spirometry results in 30 children with CF. They found a significant negative correlation between the raw IOS values (R5, Z, and Fres) and spirometry parameters. In their research, correlations were insignificant when percent predicted values were analyzed. Raj et al. (4) found a significant moderate negative correlation between IOS parameters (R and percent predicted) and spirometric parameters. X5 and R20 were weakly correlated with spirometric parameters. The authors attempted to identify IOS breakpoints to identify patients with lung dysfunction at FEV1 <40, 60, and 80%. Discriminatory power for Z5% and R5% was high at all FEV1 cutoffs (4).

In the already cited study of Moreau et al. (11) determining the relationship between IOS data and spirometry results in children with CF aged 4–19 years, it was observed that IOS measurements presented an insufficient sensitivity to detect and follow bronchial obstruction in patients with CF, which we also observed. What is more, in the case of both IA and IB subgroups, spirometric parameters correlated with LCI to a greater extent than IOS parameters. Our research also showed that diagnosis of obstruction FEV1/FVC z-score ≤ −1.64 suggests that LCI no longer correlates as strongly with changes in the FEV1/FVC z-score parameter. However, in children without diagnosed obstruction (FEV1/FVC > −1.64), the mean LCI was lower than in children with obturation. What is more, in this case, LCI coefficient correlated with FEV1/FVC z-score parameter. Our research carried out in a pediatric group of patients with CF suggests that there is a moderate significant negative correlation between LCI and selected IOS parameters (R5%pred, X5%pred, R5, R5%pred, R20, and R20%pred).

It is known that patients with CF have reduced lung compliance due to parenchymal lung disease in addition to airflow limitation (34). However, large amounts of sputum accumulated in the airways of patients with CF may hinder measurements, especially IOS. For this reason, the IOS technique may be more useful in conditions such as asthma and chronic obstructive pulmonary disease (8, 35). The findings of a review prepared by Galant et al. (36) suggest that IOS can add value to traditional clinical and spirometric assessment and thus improve management of asthma in children and adults, as well as have the potential to detect early dysfunction of the peripheral airways, which may result in better outcomes. In another trial, it was suggested that IOS and tomography could be used safely in early detection of impairment of lung function, but authors concluded that further studies are needed to evaluate the utility of IOS in clinical monitoring of children with CF who are not compliant with spirometry maneuvers (10).

## Conclusions

The described pulmonary function tests correlate similarly with spirometry, which is considered the gold standard. The presented research results suggest that the MBNW test detects disturbances in gas flow and distribution more than IOS. In the case of obstructive diseases such as asthma, the IOS technique seems to be a good diagnostic tool; nevertheless, in the case of CF, it seems justified to use it as a supplementary test. What is more, based on current evidence, we should remember that spirometry cannot be substituted by oscillometry and also by MBNW in monitoring the respiratory status of children and adolescents with CF. However, those techniques could be complementary to each other.

## Data Availability Statement

The raw data supporting the conclusions of this article will be made available by the authors, without undue reservation.

## Ethics Statement

Ethical review and approval was not required for the study on human participants in accordance with the local legislation and institutional requirements. Written informed consent to participate in this study was provided by the participants' legal guardian/next of kin.

## Author Contributions

All authors listed have made a substantial, direct, and intellectual contribution to the work and approved it for publication.

## Conflict of Interest

The authors declare that the research was conducted in the absence of any commercial or financial relationships that could be construed as a potential conflict of interest.

## Publisher's Note

All claims expressed in this article are solely those of the authors and do not necessarily represent those of their affiliated organizations, or those of the publisher, the editors and the reviewers. Any product that may be evaluated in this article, or claim that may be made by its manufacturer, is not guaranteed or endorsed by the publisher.
